# Nonadiabatic Effects in the Molecular Oxidation of Subnanometric Cu_5_ Clusters

**DOI:** 10.1021/acs.jpca.1c07271

**Published:** 2021-10-11

**Authors:** Alexander O. Mitrushchenkov, Alexandre Zanchet, Andreas W. Hauser, María Pilar de Lara-Castells

**Affiliations:** † MSME, UPEC, CNRS, Université Gustave Eiffel, F-77454, Marne-la-Vallée, France; ‡ Instituto de Física Fundamental (AbinitSim Unit), CSIC, Serrano 123, 28006 Madrid, Spain; § Institute of Experimental Physics, Graz University of Technology, Petersgasse 16, 8010 Graz, Austria

## Abstract

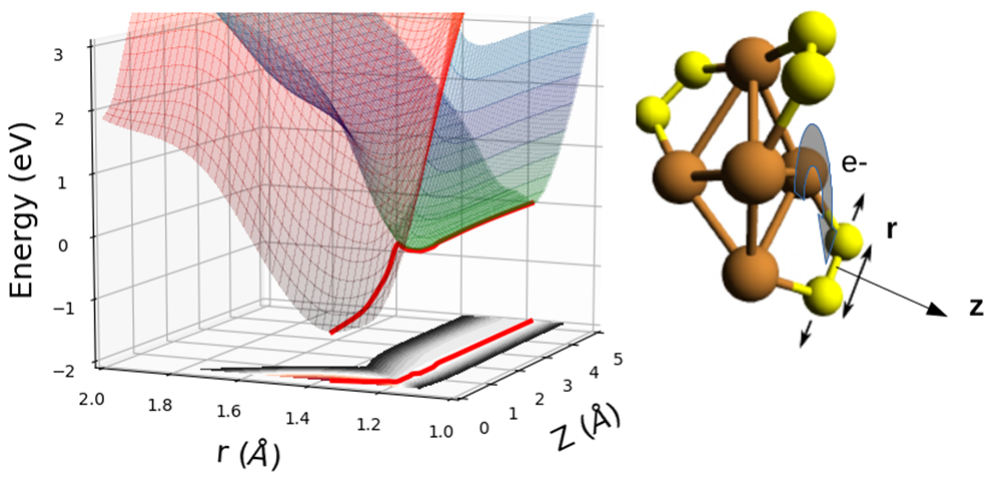

The electronic structure of subnanometric clusters, far off the bulk regime, is still dominated by molecular characteristics. The spatial arrangement of the notoriously undercoordinated metal atoms is strongly coupled to the electronic properties of the system, which makes this class of materials particularly interesting for applications including luminescence, sensing, bioimaging, theranostics, energy conversion, catalysis, and photocatalysis. Opposing a common rule of thumb that assumes an increasing chemical reactivity with smaller cluster size, Cu_5_ clusters have proven to be exceptionally resistant to irreversible oxidation, i.e., the dissociative chemisorption of molecular oxygen. Besides providing reasons for this behavior in the case of heavy loading with molecular oxygen, we investigate the competition between physisorption and molecular chemisorption from the perspective of nonadiabatic effects. Landau–Zener theory is applied to the Cu_5_(O_2_)_3_ complex to estimate the probability for a switching between the electronic states correlating the neutral O_2_ + Cu_5_(O_2_)_2_ and the ionic O_2_^–^ + (Cu_5_(O_2_)_2_)^+^ fragments in a diabatic representation. Our work demonstrates the involvement of strong nonadiabatic effects in the associated charge transfer process, which might be a common motive in reactions involving subnanometric metal structures.

## 1 Introduction

From a chemical point of view the reactivity of molecular oxygen is a complex phenomenon. In the gas phase, the ground state of O_2_ is a triplet as the molecule holds two unpaired electrons. However, when reacting with another species M such as a molecule, a metal cluster, or an extended metal-oxide surface, the bonding is generally associated with peroxo (O_2_^2–^) or superoxo (O_2_^–^) forms of molecular oxygen.^1,2^ The latter are correlated to excited ionic states of the M–O_2_ pair, where the character of the O_2_ molecule is either of singlet or doublet spin multiplicity.^3^ For a better understanding of O_2_ reactivity, it is therefore necessary to calculate the transition along the reaction pathway between the triplet O_2_ molecule in the gas phase and its associated singlet/doublet peroxo/superoxo form when engaging in chemical bonding.

In biochemistry, a clear preference for spin-saturated closed-shell systems is observed. Reactivity with O_2_ is therefore strongly quenched, explaining the stability of organic matter in a rich O_2_ environment. Nevertheless, some proteins, e.g., members of the oxygenase family, are capable of catalyzing the reduction of molecular oxygen. Proteins of this kind typically contain a transition metal cofactor that mediates the spin transition. It allows them to activate molecular oxygen and to initiate reactions such as peroxidation or hydroxilation, which are essential in certain steps of biological synthesis.^4^ Because of their high catalytic activity, oxygenases are widely used in the pharmaceutical industries and play a crucial role in the synthesis of drugs or drugs precursors.^5^ In this work, we have chosen Cu_5_ clusters as a nonorganic, industrially accessible equivalent to a biochemistry-inspired transition metal cofactor.

Subnanometric metal clusters have recently emerged as innovative materials for numerous applications in, for example, energy conversion, catalysis^6−11^ and photocatalysis.^11,12^ Moreover, the deposition of small copper and silver clusters onto TiO_2_ surfaces has proven useful for the creation of novel visible-light photoactive materials,^12,13^ potential photocatalysts for CO_2_ reduction,^11,14^ and two-dimensional (2D) polaronic materials.^14,15^ With regard to bio-oriented applications, the chemical and thermodynamical stability of Cu_5_ clusters in solution over the whole pH range is particularly promising.^16^

Unlike biological systems, which require specific catalytic proteins to reduce molecular oxygen, the Cu_5_ cluster is a doublet spin species which can combine with the triplet ground state of O_2_ to form spin states of doublet as well as quartet multiplicity, thus facilitating spin transitions. To validate its capability as a reducing agent for O_2_, the interaction between Cu_5_ and O_2_ needs to be studied in greater detail. Because Cu_5_ mediates both singlet and triplet states of O_2_, we can expect that the reactivity between those species involves several electronic configurations and can thus be considered as an, inherently, multireference problem. Their energetic proximity further suggests the presence of significant nonadiabatic effects. This implies that single-reference density functional theory (DFT) or post-Hartree–Fock approaches such as second-order Møller–Plesset (MP2) and coupled-cluster [e.g., CCSD(T)] methods can only deliver an incomplete picture of the reaction mechanism at best,^17^ making the application of multireference methods mandatory.

A first evaluation of the Cu_5_ catalytic performance employing multireference techniques was performed in ref (18). Despite the well-known tendency to complete oxidation as it is generally observed for Cu nanoparticles, *ex situ* experimental measurements^6^ and theoretical studies considering the adsorption of a single oxygen molecule^6,18,19^ have indicated an unexpected resistivity to the “irreversible” oxidation at temperatures below 423 K for Cu_5_, in both structural motifs identified by theory, i.e., a planar trapezoidal as well as a bipyramidal structure. In the latter case, the O_2_ molecule can be easily chemisorbed due to a low barrier to access the well, but the splitting of the O–O bond remained the rate-determining step for an irreversible oxidation to occur.^18^ Interestingly, in the case of a single O_2_ molecule, the chemisorption well remains relatively shallow, and because the barrier is low, the O_2_ molecule may have the possibility to desorb under the right conditions, suggesting the possibility of a “reversible” oxidation.

In this work, the application of multireference *ab initio* theory instead has allowed us to calculate the energy barriers to molecular and dissociative chemisorption when multiple O_2_ molecules become adsorbed. In fact, the high symmetry of the bipyramidal isomer of Cu_5_ clusters suggests that more than one molecule can be chemisorbed. For instance, previous studies have shown that reduced metal-oxide surfaces are capable of engaging three O_2_ molecules per vacancy (in both neutral and reduced molecular forms) at full coverage.^2^ On the other hand, the possible role of nonadiabatic effects on the oxidation of Cu_5_ clusters, and more generally of subnanometric metal clusters, in an O_2_-rich environment remains an open question. We consider the particular case of the interaction of a third O_2_ molecule with a copper cluster bearing already two O_2_ molecules, or, in other words, the O_2_ + Cu_5_(O_2_)_2_ reaction, and apply multireference *ab initio* theory within a diabatic representation. The consideration of the particular Cu_5_(O_2_)_3_ system has been motivated by the compensated loading of three O_2_ molecules on alternate Cu–Cu bonds.

The article is structured as follows. In section 2 we provide a detailed description of the computational methods. Results are presented in section 3, which includes an extended analysis of the electronic structure of the Cu_5_(O_2_)_3_ system at the molecular chemisorption state of the diabatic potential energy surfaces correlating with ionic [O_2_^–^ + (Cu_5_(O_2_)_2_)^+^] and neutral [O_2_ + Cu_5_(O_2_)_2_] fragments for the O_2_ + Cu_5_(O_2_)_2_ bimolecular reaction and of the probability of charge transfer within the Landau–Zener model. Our findings are summarized and conclusions are presented in section 4.

## 2 Computational Methods

### 2.1 Electronic Structure Calculations

Multireference perturbation theory has been applied to determine the reaction pathway of a single O_2_ molecule interacting with the Cu_5_(O_2_)_2_ complex, i.e., a Cu_5_ cluster with two O_2_ molecules adsorbed. A trigonal-bipyramidal (3D) structure has been assumed for the Cu_5_ cluster. The geometry optimization of the Cu_5_(O_2_)_3_ cluster geometry has been performed at the PBE-D3 level^20−22^ by using the ORCA^23^ suite of programs (ver. 4.0.1.2) and the atom-centered def2-TZVP^24^ basis set for copper and oxygen atoms. The optimized geometry calculated at the DFT-D3 level has been used as an initial guess in follow-up calculations with multireference *ab initio* theory. At the minima, also a single-reference DFT description can offer sufficient accuracy.

We have considered the electronic state correlating with the neutral O_2_ and Cu_5_(O_2_)_2_ fragments in the asymptotic region (termed the neutral state) and that correlating with the ionic O_2_^–^ and (Cu_5_(O_2_)_2_)^+^ ionic systems (termed the ionic state). The combination of doublet (Cu_5_) and three triplet (O_2_) states results in doublet, quartet, sextet, and octet spin states. The doublet spin state has been considered in our calculations as it is energetically favored at the molecular chemisorption state. However, since the three adsorbed O_2_ molecules, with each carrying an unpaired electron, are localized at a larger distance to each other, the energy differences between states with different multiplicity are very small.

As mentioned above, the initial structure for the chemisorption state of the Cu_5_(O_2_)_3_ complex has been the minimum-energy geometry obtained at the DFT-D3 level. The actual reaction pathway has been approximated by considering the more-involved nuclear degrees of freedom. In particular, in the given scenario, the optimized internuclear O–O distance has been found to differ significantly in neutral and ionic states at the corresponding physisorption and molecular chemisorption energy minima. Therefore, to obtain a sensible estimation of the actual reaction pathway, we have performed a two-dimensional (2D) scan: The first coordinate (*Z*) is the distance of the oxygen molecule (its center of mass) to the cluster, with *Z* = 0 defined as its optimized position in the chemisorption minimum of the Cu_5_(O_2_)_3_ complex. The second coordinate is the internuclear O–O distance (*r*). The remaining degrees of freedom have been kept fixed in these 2D scan calculations. We have applied the single-reference internally contracted RS2C method^25^ with density fitting (DF-RS2C), as implemented within the MOLPRO program package.^26^

To calculate the neutral and ionic states, we have followed the methodological strategy proposed in refs (27) and (28) for the interaction of O_2_ with a reduced TiO_2_ surface. We first optimize the orbitals by using the single-state DF-CASSCF method separately for neutral and ionic states, which allows to calculate directly diabatic states.^28^ Because neutral and ionic states bear a very different electronic character, the corresponding optimized orbitals for these two states are also very different. Hence, common approaches using a common set of orbitals would require very large configuration interaction (CI) expansions, and the calculation would be computationally unfeasible. The use of orbitals optimized separately for each state is thus natural. To ensure continuous behavior of the electronic structure along *Z*, we start the calculation at large distance, where the neutral oxygen molecule (and ion) are well separated from the copper cluster. Starting from this distance, we move inward, repeatedly using the electronic wave function of the previous step as a starting guess and ensuring that the orbitals are only modified gradually. At present, this is possible by using the SUPER-CI optimization method^29^ as implemented in a recent version of the MOLPRO code.^30^ The DF-CASSCF approach is employed to account for the most important nondynamic correlation effects. Then, the single-reference RS2C method is applied to cover dynamical correlation effects. Because the two diabatic states (ionic and neutral) can cross, it is easy to locate the seam in the 2D representation depending on (*r*, *Z*). The location of the transition state between physisorption and chemisorption states corresponds to the minimum energy crossing point (MECP) between these two diabatic states, being thus given by the position (*Z*, *r*) of the minimum of the seam. Similarly to the case of the O_2_–TiO_2_ system (see refs (27 and 28)), neutral and ionic states are effectively diabatic but nonorthogonal states. The overlap and the transition matrix element of the electronic Hamiltonian between these two states are calculated at the CASSCF level by using the transformation to biorthogonal orbitals as implemented within MOLPRO.^31^ As the two states are nonorthogonal, the unique definition of diabatic states is not possible as it depends on the used orthogonalization method (e.g., symmetric vs Schmidt). Therefore, a transformation to adiabatic states is performed to estimate the effective nonadiabatic interaction. After orthogonalization, the transformed 2 × 2 electronic Hamiltonian is diagonalized to obtain the adiabatic energies that form the avoided crossing. Next, H_12_ can be calculated as the half of the energy difference between the two adiabatic surfaces at the point when this difference is minimal (at the CASSCF level). This value of H_12_ is used together with diabatic energies obtained at the DF-RS2C level.

To carry out the DF-CASSCF/DF-RS2C calculations, we use the polarized correlation-consistent triple-ζ basis of Dunning and collaborators^32^ (cc-pVTZ) for oxygen atoms and the cc-pVTZ-PP basis set for copper atoms,^33^ including a small (10-valence-electron) relativistic pseudopotential. For density fitting, the associated MP2FIT and JKFIT bases were used in CASSCF and RS2C calculations, respectively. For the 2D scans of interactions energies in neutral and ionic states, the active space was built from 7 electrons in 7 orbitals (referred to us as (7 7) active space), while 68 closed-shell orbitals were fully optimized at the DF-CASSCF level. Of these, 32 core orbitals were not correlated at the DF-RS2C level. To get an estimation of the energy barrier to O_2_ dissociation, it was necessary to increase the active space [13 electrons in 11 orbitals, referred to us as (13 11) active space] due to the higher multiconfigurational character of the wave function upon the O–O bond elongation. Additionally, the whole Cu_5_(O_2_)_3_ structure at the chemisorption minimum was reoptimized at the CASSCF level by using the (13 11) active space, with the optimized *r* value being 1.273 Å. Next, the *r* value was varied, keeping the rest of the coordinates fixed to the corresponding optimized values at the CASSCF-derived optimized geometry, thus obtaining a potential energy curve as a function of *r*.

The diagonalization of the first-order reduced dentity matrix obtained from the wave function in CASSCF calculations allows to obtain natural orbitals. The chemical oxidation states of the copper atoms as well as the reduced forms of the adsorbed O_2_ molecules in the Cu_5_(O_2_)_3_ complex have been deduced from an analysis of the Mulliken charges^34^ and atomic spin populations of the CASSCF-derived natural orbitals with the Hirshfeld method.^35,36^

### 2.2 Landau–Zener Model

To estimate the electron hopping probability between the diabatic electronic states of the O_2_–Cu_5_(O_2_)_2_ complex correlating with neutral and ionic fragments, we apply the Landau–Zener (LZ) model.^37−39^ Within the LZ model, the electron hopping probability can be defined as 1 – *P*_LZ_, with *P*_LZ_ being the LZ probability for a nonadiabatic transition:
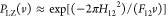
1In eq 1, *v* is the relative velocity of the fragments, *F*_12_ is the difference between the two slopes *F*_1_ and *F*_2_ of the diabatic potential energy surfaces at the intersection between the neutral and ionic states, and *H*_12_ is the off-diagonal matrix element of the electronic Hamiltonian. Assuming a Maxwell–Boltzmann (MB) distribution for the relative velocities of the reactants, the electron hopping probability can be written as a function of temperature. For this purpose, we integrated over the hopping probabilities from the LZ model, *P*_LZ_, expressed as a function of the velocity *v* in the reaction coordinate (i.e., defining the minimum-energy pathway), and weighted with a Boltzmann factor *P*_MB_
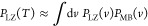
2where *P*_MB_(*v*) denotes the MB distribution of relative velocities in one direction
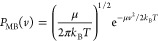
3with *k*_B_ as the Boltzmann constant.

## 3 Results and Discussion

### 3.1 Chemisorption Minima. Oxidation States of Cu Atoms

Reactivity with molecular oxygen is generally associated with strong electronic correlation effects, making the assignment of oxidation–reduction states complicated in high level *ab initio* calculations. In this sense, one key advantage of the diabatic representation is that the character of the wave function is preserved along the reaction path (see, e.g., ref (28)). This feature can be used as an useful interpretative tool to identify the oxidation–reduction states, particularly in the case of O_2_. Indeed, in the asymptotic region, the two fragments [i.e., O_2_ and Cu_5_(O_2_)_2_] are not interacting. Hence, the simple inspection of the orbital occupation numbers allows to determine the states corresponding to neutral and ionic interacting pairs. By following these states diabatically along the reaction path, it is possible to assign the correct reduction state. The region of electron transfer corresponds to the crossing between ionic and neutral states.^3,28^

**Figure 1:**
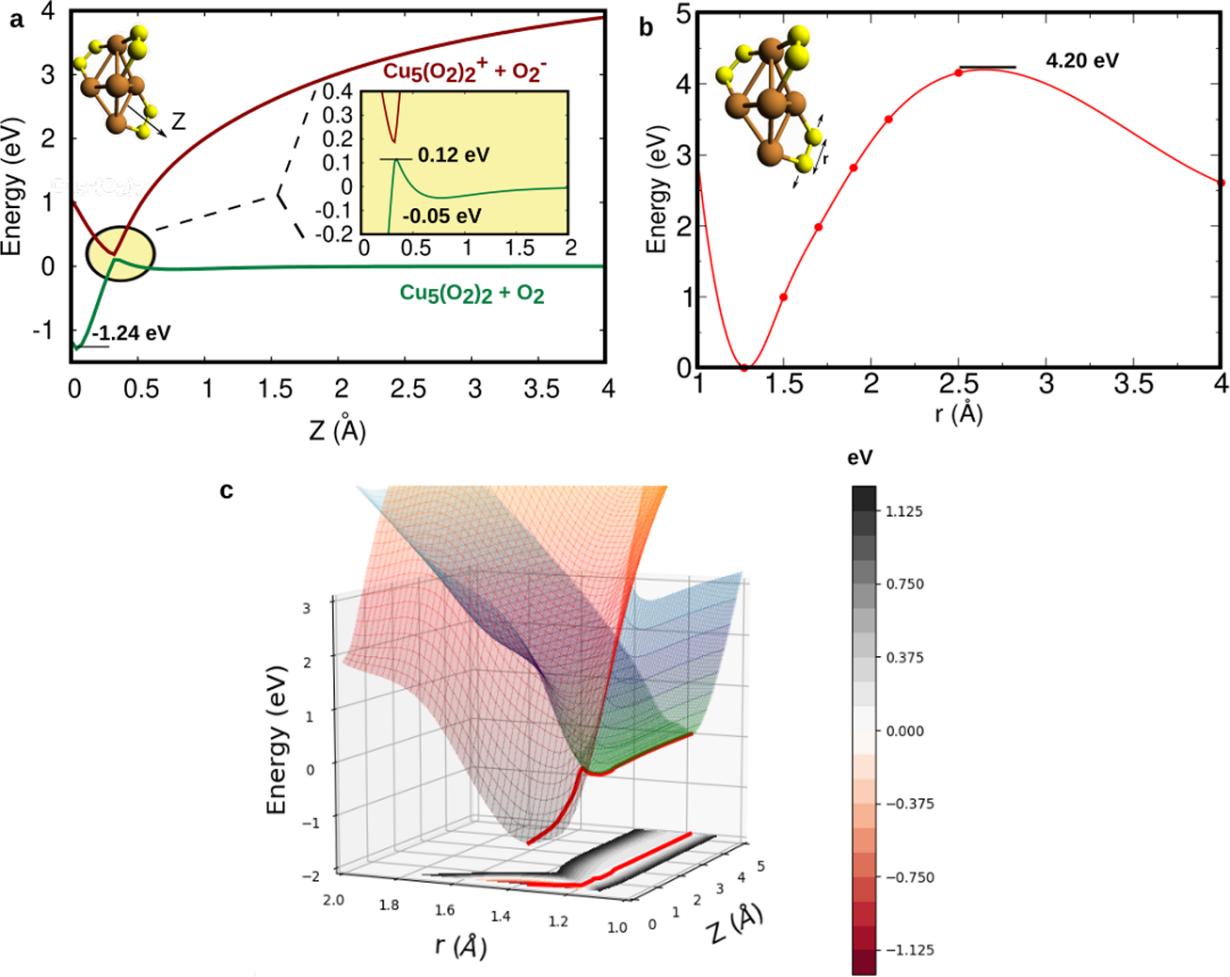
(a) O_2_ + Cu_5_(O_2_)_2_ reaction energy pathway in the adiabatic representation using the (7 7) active space. In the reactants region, the neutral state (green) corresponds to the ground state and the ionic pair state (red) corresponds to the excited state. In the chemisorption region, the ground state present ionic character while the neutral state lies higher in energy. For clarity, an expanded view of the region of the barrier is represented showing the avoided crossing between the two states. (b) Potential energy scan providing the energy dependence upon the O–O stretching of one chemisorbed O_2_ molecule by using the (13 11) active space. The figure shown in (a) corresponds to a 2D scan in the *r* and *Z* coordinates, with the rest of the coordinates having been kept fixed. The *Z* coordinate in the potential energy curve shown in (b) has been kept fixed instead, with the energies having been given relative to the value obtained at *r* = 1.273 Å. (c) Two-dimensional (2D) representation of the diabatic potential energy surfaces (PES), including the minimum-energy pathway. Contour plot of the ground adiabatic PES and its energy scale are also shown for clarity.

**Figure 2:**
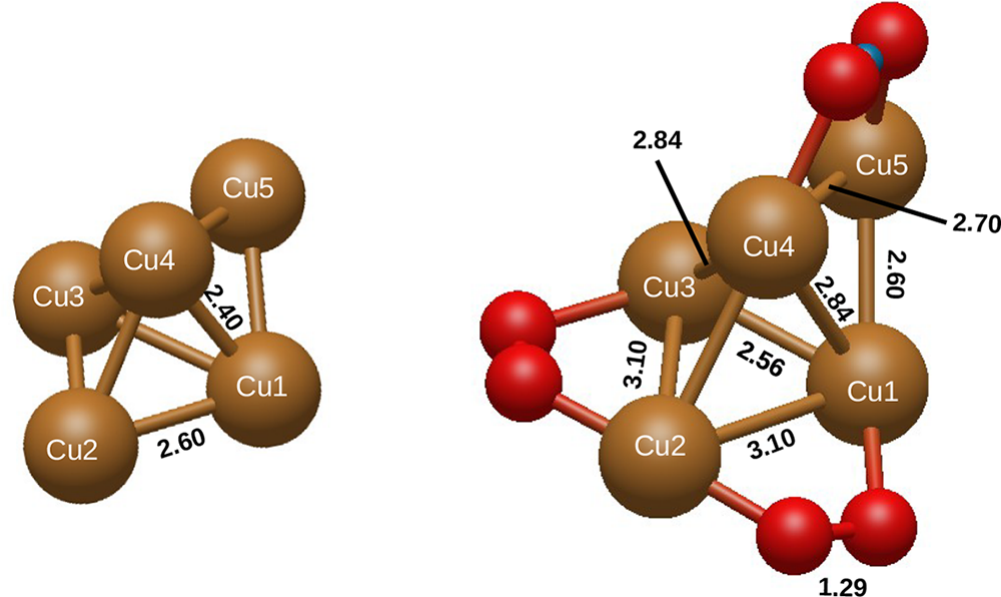
Geometry of the bare Cu_5_ cluster (left panel) and the Cu_5_(O_2_)_3_ complex (right panel). Values of internuclear distances are given in angstroms. Symmetry-equivalent Cu–Cu distances have been skipped in the figure (e.g., the value of the Cu1–Cu3 distance in the panel on the left is 2.40 Å due to the symmetry of the trigonal-bipyramidal Cu_5_ structure).

It can be observed in Figure 1 that the chemisorption well of the Cu_5_(O_2_)_3_ complex correlates directly with the ionic O_2_^–^ + (Cu_5_(O_2_)_2_)^+^ interacting pair. This implies that the incoming oxygen molecule captures an electron from the Cu_5_(O_2_)_2_ complex, becoming chemisorbed as a superoxo ion O_2_^–^. A Mulliken charge analysis of the natural orbitals can be applied to determine the reduction state of the remaining two O_2_ molecules. The analysis of the more stable structure shown in Figure 2 reveals that the three O_2_ molecules of the Cu_5_(O_2_)_3_ complex at the chemisorption minimum bear a similar charge of ca. −0.7 |*e*|. It is known that the Mulliken analysis tends to underestimate the degree of ionicity but remains globally proportional to the real charge distribution. Because the incoming O_2_ molecule becomes absorbed as a superoxo O_2_^–^ radical, it follows that all the three O_2_ molecules are attached to the copper cluster as O_2_^–^ species, with each of them having taken one electron from the Cu_5_ cluster.

The assignment of reduction states is further confirmed by the analysis of spin atomic populations, which indicates the existence of three unpaired electrons when three O_2_ molecules become chemisorbed onto the copper cluster. From the analysis of the multireference wave function, it is also clear that all O_2_ molecules in the Cu_5_(O_2_)_3_ complex can be characterized by a spin of almost unity, confirming the superoxo O_2_^–^ radical nature of the three chemisorbed O_2_ molecules.

The conversion of the three O_2_ molecules in superoxo species is also reflected in the enlargement of the O–O bond lengths (larger than ca. 1.3 Å) upon from the value for the neutral O_2_ molecule (ca. 1.2 Å). The inclusion of the dispersion interaction via the D3 *ansatz*(21) in the DFT-based description is in fact correlated to the enlargement of the Cu–Cu bonds, as necessary to accommodate the superoxo species with larger O–O bond lengths than for the neutral O_2_ molecule. The reoptimization of the structure using the D4 Grimme’s parametrization^40^ modifies the Cu–Cu bond length by less than 10^–3^ Å.

**Figure 3:**
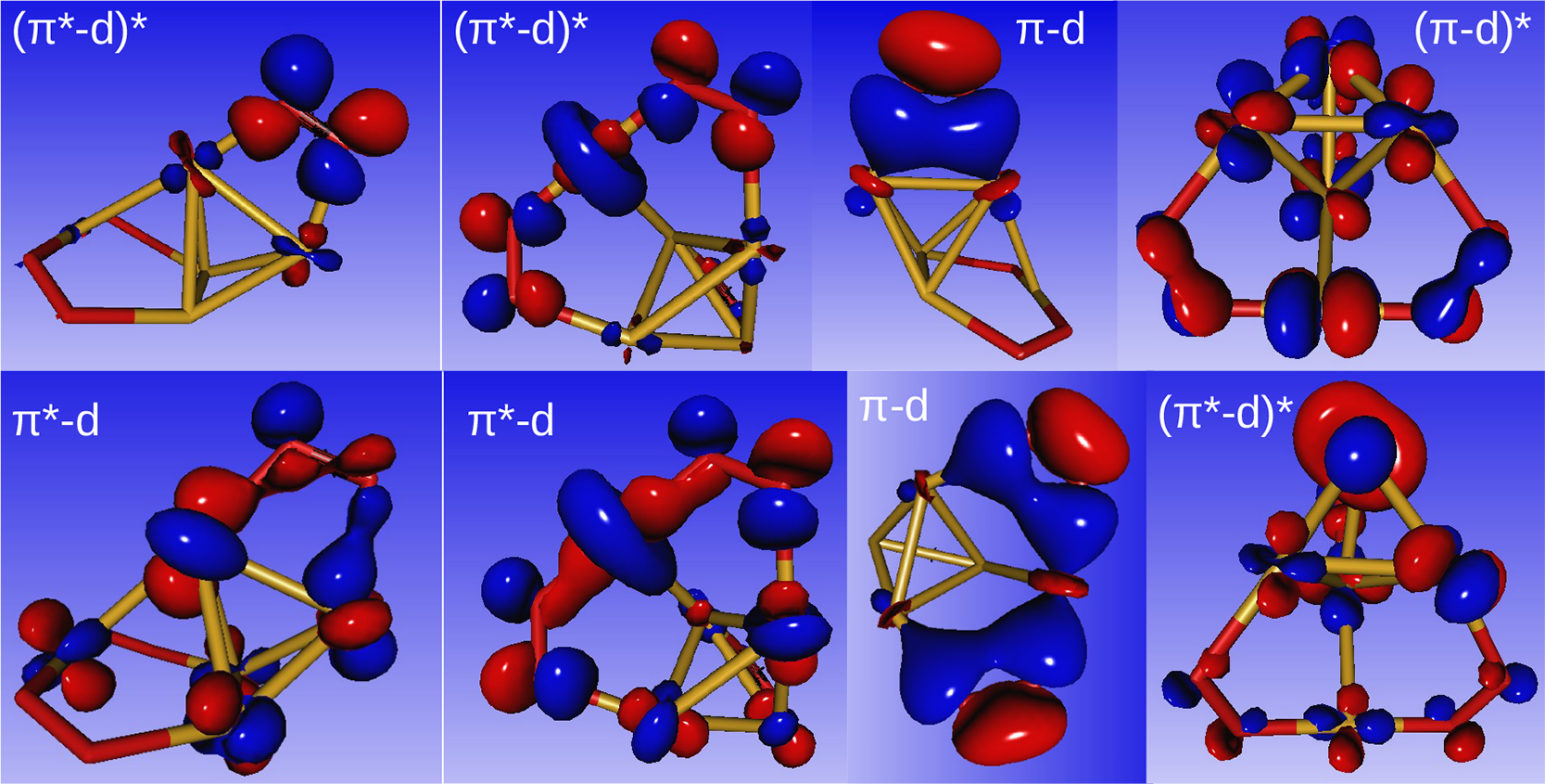
Main (natural) orbitals involved in the bonding between the Cu_5_ cluster and the three adsorbed O_2_ molecules in the Cu_5_(O_2_)_3_ complex. Note the pronounced bonding between 3d(Cu) and π(O_2_) orbitals.

Having confirmed the reduction state of the chemisorbed oxygen molecules, it is also possible to extract the individual oxidation numbers for the copper atoms from the Mulliken charge analysis. The copper atoms labeled as Cu1, Cu3, Cu4, and Cu5 in Figure 2 bear a positive charge of 0.35 |*e*| each. In contrast, the copper atom labeled Cu2, located between the two O_2_ molecules, has a positive charge of 0.7 |*e*|, which is identical in size to the negative charges assigned to the O_2_ molecules. It can therefore be concluded that the Cu2 atom exhibits a net positive charge, while the remaining positive charges are delocalized over the other four copper atoms. We note that the Cu2 atom differs from the others in being the only atom bonded to two O_2_ molecules. On average, half an electron is provided from each Cu atom to the bonded oxygen atom. Such partial values of the charges are indicative of a concerted, collaborative behavior between all Cu atoms for charge transfer, which is attributed to a complex metal–ligand bonding scheme involving the d orbital network of the Cu_5_ cluster with both the π and π* orbitals of the adsorbed O_2_ molecules, as can be readily seen in Figure 3.

The modification of the Cu_5_ electronic structure and geometry upon adsorption of three molecules compared with that of the bare Cu_5_ cluster invokes the concept of structural fluxionality of metal clusters at the subnanometric scale, a feature with the potential to enhance its catalytic activity^11^ (see also, e.g., ref (41)). Of course, many local minima associated with different positions of the three adsorbed O_2_ molecules might have similar energies to that calculated for the global minimum at the chemisorption state. Such energy landscape is implicitly associated with fluxional dynamics and wide amplitude motions in the Cu_5_(O_2_)_3_ complex where the O_2_ molecules can readily exchange positions. An important role of structural fluxionality has been recently advocated for Ni_5_ clusters having a trigonal-bipyramidal 3D structure with an intruder Au atom, enhancing the weight of the configurational entropy.^42^ The occurrence of a fluxional dynamics on cationic Cu_5_^+^ clusters has been also suggested from recent IR spectroscopic characterizations of H_2_ adsorption.^43^

### 3.2 Potential Energy Surfaces in a Diabatic Representation

As mentioned in the Computational Methods section, the neutral and ionic states characterizing the O_2_–Cu_5_(O_2_)_2_ interaction are calculated in a similar way to that employed in a previous study of the “molecular” oxidation of a reduced TiO_2_(110) surface (see ref (28)). The ionic state, which is the ground state at the chemisorption minimum, is characterized by a strong Coulombic interaction between a negatively charged superoxo O_2_^–^ radical and a positively charged (Cu_5_–(O_2_)_2_)^+^ system. It is asymptotically correlated to a separation in charged fragments O_2_^–^ and (Cu_5_(O_2_)_2_).^+^ In contrast, the neutral state corresponds to the interaction between neutral fragments and leads to a separation into an oxygen molecule and the Cu_5_(O_2_)_2_ complex. This state is the ground state at the physisorption minimum, as shown in Figure 1a. The wave function at the transition state (TS) geometry between chemisorption and physisorption shows a strong multiconfigurational character. The same holds for the TS geometry at O_2_ dissociation, which reconfirms the necessity of a multireference method for an appropriate analysis of the reaction pathway. A barrier height of 0.12 eV is obtained, which is comparable to that published in ref (18) (0.09 eV), where only one O_2_ molecule was considered for the first step of the oxidation of Cu_5_. It is interesting to observe that chemisorbed O_2_ molecules are barely affecting the height of the barriers in successive molecular oxidation steps.

In contrast, an interesting collaborative effect involving delocalized d-type orbitals of the Cu atoms is found with regards to the stability of the chemisorption well, which is much deeper (−1.16 eV) in the case of the O_2_ + Cu_5_(O_2_)_2_ reaction than in the first oxidation step of the Cu_5_ cluster. As can be observed in Figure 1a, despite this collaborative effect, due to the high energy of the excited state correlating with the O_2_^–^ + (Cu_5_(O_2_)_2_)^+^ fragments, the oxygen molecule is released as a neutral molecule. In other words, a net charge on the cluster oxide Cu_5_(O_2_)_2_ as an independent species is energetically forbidden: it loses an electron when the O_2_ molecule is adsorbed but recovers it upon O_2_ desorption. This is a direct consequence of the high ionization potential exhibited by the subnanometric copper cluster.

To ensure that the oxidation is indeed reversible, in addition to discarding the possibility of chemical ionization, it is further necessary to check whether the O_2_ molecules can dissociate or not. In fact, because the collaborative effect induces a larger stability than a single O_2_ molecule, one may wonder if this extra energy allows the O_2_ molecule to dissociate on the cluster. Figure 1b indicates that it is not the case. The estimated value of the energy required to dissociate O_2_ is higher than 4 eV, much larger than the extra stability provided by the collaborative effect and very similar to the case of single O_2_ adsorption.^18^ The reason for the estimated high value of the energy barrier to O_2_ dissociation is attributed to the complex binding network formed between the O_2_ molecules and the Cu_5_ clusters. By relaxing the whole Cu_5_(O_2_)_3_ complex at the *r* value associated with the TS state (2.5 Å), the energy barrier becomes lower by only 0.2 eV at the CASSCF level. Therefore, it is improbable that even considering higher levels of *ab initio* theory, it gets flat enough to render the breaking of the O_2_ bond kinetically allowed at room and experimentally relevant temperatures.

An illustration of the collaborative effect mentioned above is given in Figure 3. The very favorable overlap between the π and π* orbitals of O_2_ with d-type orbitals of Cu_5_ gives rise to a complex and stable π–d−π* molecular orbital network. Interestingly, not only the frontier orbitals are involved, but also π-type and inner d-type orbitals are contributing to the chemical engagement between copper and oxygen atoms. To break the O–O bond, in addition to the intrinsic dissociation energy of molecular oxygen, it would be necessary to provide additional energy input to fragment this complex π–d−π* orbital network. The involvement of π* orbitals is a classic case of back-bonding and an intermediate state of O–O bond dissociation. However, the fact that the usual electron transfer from bonding into antibonding orbitals is coupled to the sterically less flexible Cu_5_ orbital network makes the O–O bond rupture energetically more costly.

### 3.3 Landau–Zener Model

**Figure 4:**
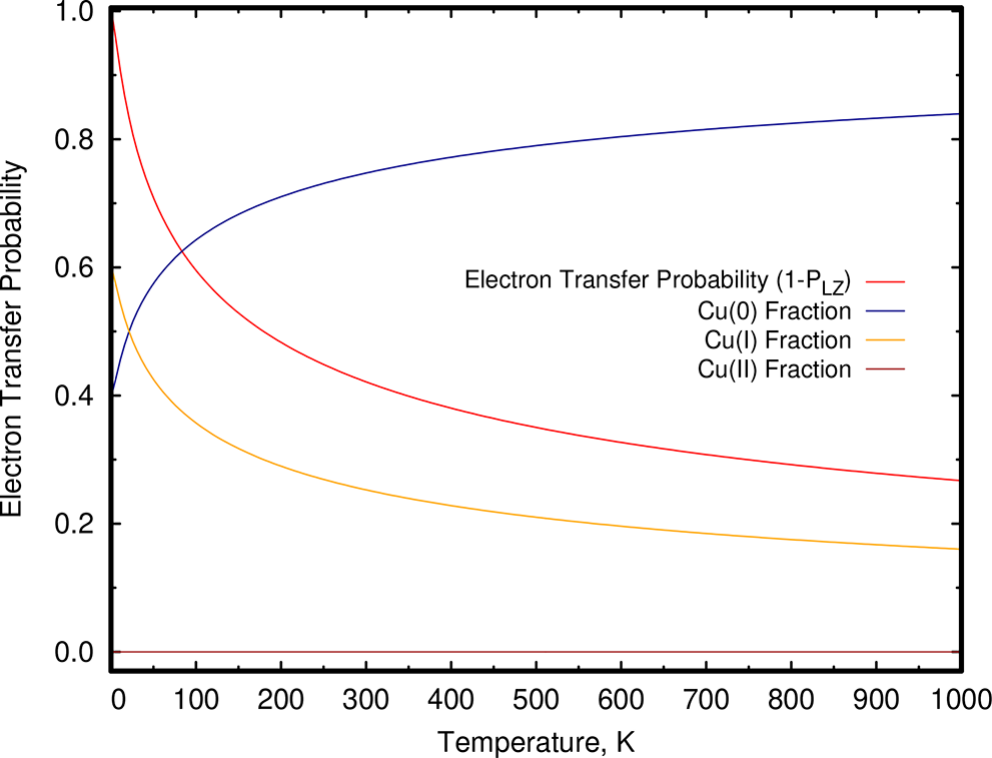
Electron hopping probability as a function of the kinetic temperature in the reaction coordinate. Probability of electron hopping (1 – *P*_LZ_) from the copper cluster of the Cu_5_(O_2_)_2_ complex to an incoming O_2_ molecule within the Landau–Zener model for nonadiabatic transitions.^39^ The fraction of the oxidation copper state Cu(I) is also shown. It has been estimated as (1 – *P*_LZ_) × *f*_Cu(I)_, with *f*_Cu(I)_ the fraction of the Cu(I) state in the Cu_5_(O_2_)_3_ complex (calculated at the same level of theory as the diabatic electronic states).

Within the Landau–Zener model, the probability for electron hopping from the Cu_5_(O_2_)_2_ complex to an incoming O_2_ molecule is predicted to decrease—and thus the Cu(I) proportion—as the temperature increases (see Figure 4). This outcome can be interpreted as follows: On one hand, similarly to a charge transfer harpoon-type reaction,^39^ lower temperatures imply lower relative velocities of the reactant species (i.e., the incoming O_2_ molecule and the Cu_5_(O_2_)_2_ complex). On the other hand, lower velocities favor the electron hopping process because more time is spent at the crossing region between the diabatic electronic states correlating with ionic and neutral fragments. Because of the smaller value of the H_12_ as compared to the case of the Cu_5_–O_2_ complex (see ref (18)), the probability for the Cu_5_(O_2_)_3_ complex to remain neutral as the temperature increases becomes much larger. As discussed in ref (18), the LZ probability of nonadiabatic transitions for the O_2_ + Cu_5_ system is very small so that the height of adiabatic energy barrier from physisorbed to molecular chemisorbed states determines accessibility of the latter at a given temperature. For the Cu_5_ cluster having a 3D structure, such a barrier is very small (0.09 eV, see ref (18)) so that the chemisorption state is accessible even at room temperature. In stark contrast, the barrier for the Cu_5_ cluster having a planar structure is very large, avoiding accessibility at room and higher temperatures.^18^ Hence, our results indicate the enhancement of nonadiabatic effects upon adsorption of multiple O_2_ molecules on the Cu_5_ cluster. Although the LZ model has just been applied to a particular collision event, it might be representative of the physics occurring at ultrahigh vacuum (UHV) when molecular beams of O_2_ are scattered by Cu_5_ clusters (i.e., when the kinetic regime of bimolecular collisions is dominating).

Summarizing this section, by including nonadiabatic effects via the LZ model, the probability of electron hopping from the Cu_5_(O_2_)_2_ complex to an incoming O_2_ molecule is predicted to decrease—and then the proportion of the oxidated Cu(I) form—as the (LZ) temperature increases since, as mentioned above, higher velocities reduce the time spent in the crossing region between the diabatic states correlating with ionic and neutral fragments.

## 4 Conclusions

The application of multireference perturbation theory to the O_2_–Cu_5_ system^18^ and the O_2_–Cu_5_(O_2_)_2_ complex indicates high energy barriers for O_2_ dissociation. Conversely, the energetic barriers to reach the molecular chemisorption states from the physisorption counterparts are very low (ca. 0.1 eV, see Figure 1a). Hence, the oxidation is expected to be reversible even if several O_2_ molecules are chemisorbed. From a broader perspective, our findings underline the key role of nonadiabatic effects in the charge transfer process characterizing the O_2_ + Cu_5_(O_2_)_2_ bimolecular collision. The electronic structure of the complex is strongly affected by the somewhat “floppy” character of the cluster geometry, which gives rise to pronounced nonadiabatic effects. Similar tendencies are expected to be rather general for charge transfer processes between molecular systems and subnanometric metal clusters. Temperature-dependent experimental studies, using molecular beams of O_2_ scattered by subnanometric metal clusters at ultrahigh vacuum, would provide very useful insights into the described reduction process upon heating and its underlying nonadiabatic effects. Nonadiabatic multidimensional quantum dynamics calculations on the molecular oxidation of subnanometric clusters would be also very valuable in delivering mechanistic information about the interplay between fluxional dynamics and charge transfer.

In conclusion, our results indicate that Cu_5_ clusters are industrially feasible candidates for the mediation of chemical reactions involving spin transitions such as the reduction of molecular oxygen. An extension toward biochemical applications will necessitate a further theoretical assessment of Cu_5_ in solution and in biological environments, preferably starting with a modeling of the same process in water. To render the calculation computationally feasible, the application of polarizable continuum models such as those developed by Barone and co-workers seem promising.^44−48^ Early work on nucleobases, combining hybrid HF/DFT PBE0 and time-dependent PBE0 treatments with a multiconfigurational *ab initio* description to locate conical intersections, appears particularly reassuring.^49^

## Supporting Information Available

Cluster geometries in Cartesian coordinates of the most relevant structures of the Cu_5_(O_2_)_3_ complex at the (1) the minimum-energy crossing point between ionic and neutral states, (2) the molecular chemisorption minimum, and (3) the transition state between molecular and dissociative chemisorption states (PDF)

## Notes

The data supporting the results presented in this work are available from the corresponding authors on reasonable request.
