# Supplementary material for: Nonadiabatic Effects in the Molecular Oxidation of Subnanometric Cu5 Clusters
Source: J Phys Chem A. 2021 Oct 11;125(41):9143–50. doi: 10.1021/acs.jpca.1c07271 (PMC8543446; doi:10.1021/acs.jpca.1c07271)
Supplement: Supplementary file 1 — jp1c07271_si_001.pdf [file jp1c07271_si_001.pdf]

Supporting Information for the Manuscript: "Non-Adiabatic Effects in the Molecular Oxidation of Subnanometric Cu_5 Clusters "

Alexander O. Mitrushchenkov,[†] Alexandre Zanchet,[‡] Andreas W. Hauser,[*,¶] and María Pilar de Lara-Castells[*,‡]

†*Université Paris-Est, Laboratoire Modélisation et Simulation Multi Echelle, MSME UMR 8208 CNRS 5 bd Descartes, 77454 Marne-la-Vallée, France*

‡*Instituto de Física Fundamental (AbinitSim Unit), CSIC, Serrano 123, 28006 Madrid, Spain*

¶*Graz University of Technology, Institute of Experimental Physics, Petersgasse 16, 8010 Graz, Austria.*

E-mail: Andreas.W.Hauser@gmail.com; Pilar.deLara.Castells@csic.es

This Supporting Information is organized as follows:

- **The first section** presents a figure of the active orbitals used in multireference calculations of the $Cu_5(O_2)_3$ complex at the molecular chemisorption minimum.
- **The second section** lists the Cartesian coordinates of a few relevant geometries (in Å).

S1 Active orbitals of the $Cu_5(O_2)_3$ complex at the molecular chemisorption minimum

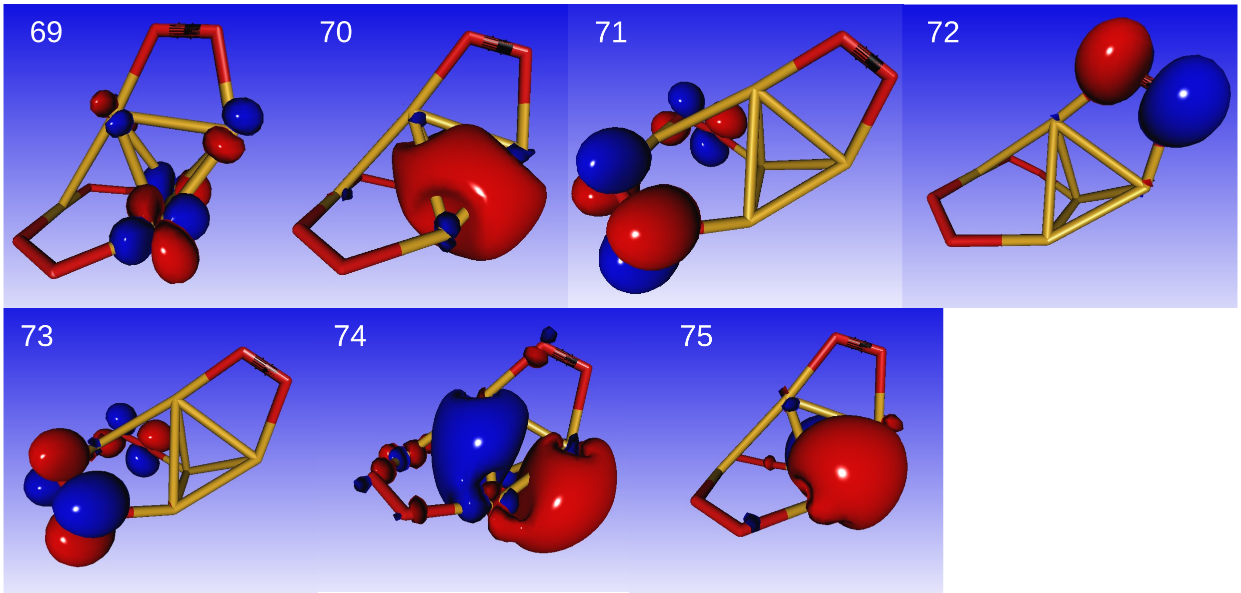

Figure S1: Molecular orbitals included in the active space of the multireference calculations for the $Cu_5(O_2)_3$ complex at the molecular chemisorption state (see the main manuscript). The orbitals 71, 72, and 73 hold an unpaired electron.

S2 Geometries of the $Cu_5(O_2)_3$ Complex

Table S1: Cartesian coordinates of the $Cu_5(O_2)_3$ complex at the transition state between physisorption and molecular chemisorption minima.

Atom	X (Å)	Y (Å)	Z (Å)
Cu	−0.02752527	−1.15955628	−1.00311002
Cu	2.02761530	0.06472133	−0.67128364
Cu	−1.94992622	−0.00838828	0.59993676
Cu	0.47059664	−0.03680053	1.20513407
Cu	−0.08040299	1.22539388	−0.93086856
O	−1.36519927	−2.42130164	−0.50715936
O	−2.10350325	−1.81762358	0.48120602
O	2.57369395	−0.07439370	2.10119857
O	3.35236428	−0.07850536	1.20814268
O	−2.23782255	1.78254419	0.47241563
O	−1.48720643	2.42816748	−0.48065957

Table S2: Cartesian coordinates of the $Cu_5(O_2)_3$ complex at the molecular chemisorption minimum.

Atom	X (Å)	Y (Å)	Z (Å)
Cu	−1.0082968370	−1.3657459447	−0.0478293937
Cu	0.5055451165	0.0507149728	2.2619846012
Cu	−1.2184840169	1.1850728634	−0.0746689899
Cu	−0.5644797463	−0.0690184553	−2.2717308393
Cu	1.3808702587	0.1225102673	−0.4197060273
O	−0.2201060967	−2.4640507787	1.5676655682
O	0.5666647334	−1.7963811919	2.3304690811
O	0.3542831879	1.8932689823	2.2416582788
O	−0.5517427206	2.4237072613	1.5021519693
O	2.2415128056	0.1752924118	−2.1860496287
O	1.2032287763	0.0718701592	−3.2636720636

Table S3: Cartesian coordinates of the $Cu_5(O_2)_3$ complex at the transition state to the dissociative chemisorption minimun.

Atom	X (Å)	Y (Å)	Z (Å)
Cu	0.81673555	−1.48767638	0.05453093
Cu	0.51261580	−0.19421301	2.26120731
Cu	−0.50545001	0.13424954	−2.19643851
Cu	−1.40718951	0.35187373	0.33566410
Cu	1.43944883	0.96515064	0.12872562
O	−0.07258337	−2.44849659	−1.57855747
O	−0.76296287	−1.68083356	−2.34237932
O	−2.53927106	0.63853066	1.73814842
O	−0.89226866	0.19937992	3.56695397
O	−0.05461611	1.91103357	−2.22342268
O	0.92122543	2.29633855	−1.47902546
